# Bactericidal and Fungistatic Properties of LDPE Modified with a Biocide Containing Metal Nanoparticles

**DOI:** 10.3390/ma14154228

**Published:** 2021-07-28

**Authors:** Katarzyna Janczak, Daria Kosmalska, Daniel Kaczor, Aneta Raszkowska-Kaczor, Lauren Wedderburn, Rafał Malinowski

**Affiliations:** Łukasiewicz Research Network—Institute for Engineering of Polymer Materials and Dyes, 87-100 Toruń, Poland; katarzyna.janczak@impib.lukasiewicz.gov.pl (K.J.); daria.kosmalska@impib.lukasiewicz.gov.pl (D.K.); daniel.kaczor@impib.lukasiewicz.gov.pl (D.K.); aneta.kaczor@impib.lukasiewicz.gov.pl (A.R.-K.); lauren.wedderburn@impib.lukasiewicz.gov.pl (L.W.)

**Keywords:** bactericidal properties, fungistatic properties, polyethylene, nano-zinc, nano-silver, nano-copper, nano-iron oxide

## Abstract

The aim of this study was to ascertain whether the combined action of metal nanoparticles (silver, copper, zinc oxide, iron oxide) would ensure the appropriate biocidal properties oflow-density polyethylene (LDPE) against pathogenic microorganisms. According to the research hypothesis, appropriately selected concentrations of the applied metal nanoparticles allow for a high level of biocidal activity of polymeric materials against both model and pathogenic bacterial strains (*Escherichia coli*, *Staphylococcus aureus*, *Pseudomonas aeruginosa*, *Legionella pneumophila*, *Salmonella enterica* subsp. *enterica*) and fungi (*Aspergillus brasiliensis*, *Saccharomyces cerevisiae*, *Candida albicans*, *Penicilium expansum*), whilst ensuring the safety of use due to the lack of migration of particles to the surrounding environment. Studies have shown that adding 4% of a biocide containing Ag, Cu, ZnO, and Fe_2_O_3_ nanoparticles is the most optimal solution to reduce the number of *S. aureus*, *S. enterica* and *P. aeruginosa* by over 99%. The lowest effectiveness was observed against *L. pneumophila* bacteria. As for *E. coli*, a higher biocide content did not significantly increase the antibacterial activity. The results showed a high efficiency of the applied biocide at a concentration of 2% against fungal strains. The high efficiency of the obtained biocidal results was influenced by the uniform dispersion of nanoparticles in the material and their low degree of agglomeration. Furthermore, a slight migration of components to the environment is the basis for further research in the field of the application of the developed materials in industry.

## 1. Introduction

The growing concern related to the emergence of newer drug-resistant strains of bacteria, fungi, and viruses that threaten human health and life is conducive to the dynamic development of materials ensuring microbiological safety. It is estimated that in the following years, among other aspects in connection with the SARS-CoV-2 coronavirus pandemic, there will be an intensive increase in the demand for antimicrobial coatings [1].

Some microorganisms, such as *Streptococcus*, *Staphylococcus*, and *Escherichia*, are responsible for nosocomial infections, which affect 2 million people per year in the United States alone, resulting in 90,000 deaths [2]. The problem of microbial infections not only affects hospitals. When deposited on item surfaces, many microorganisms can form a protective matrix of DNA, proteins, and polysaccharides called an exopolysaccharide matrix (EPS). Colonies of microorganisms surrounded by EPS are called biofilms. Destroying microorganisms after they have created a biofilm is even 1000 times more difficult [3]. Since synthetic polymer materials are subject to very slow degradation, they are usedin water, ventilation, and other systems. These are target conditions for materials’ long-term exposure to colonization by microorganisms and may constitute the focus of pathogenic microorganisms [4].

Most often, the biocidal properties of polymeric materials are tested in the presence of *Escherichia coli* and *Staphylococcus aureus* bacteria, as well as *Pseudomonas aeruginosa*, which are indicated as references in international standards [5,6,7]. These are pathogenic strains, such as the source of nosocomial infections, that sometimes mutate into drug-resistant forms, leading to serious health complications or death. One of the biological hazards associated with using microbiologically contaminated ventilation or water systems is infection with a Gram-negative strain of *Legionella pneumophila*, which causes a respiratory disease called legionellosis. It is a strain with a high survival potential in unfavorable conditions with a tendency to produce a biofilm [8]. In the food industry, a major threat to stored food is the rapidly multiplying *Salmonella enterica* subsp. *enterica*, whose source of infection is poultry and eggs [9].

Research on materials with biocidal properties seems to be focused more on action against bacteria than fungi. Meanwhile, both of them, apart from deteriorating the properties of polymeric materials, pose a serious health risk. The *Aspergillus brasiliensis* strain is one of the causative agents of bronchopulmonary aspergillosis. Most often, infection occurs through inhalation of polluted air, and less frequently through direct ingestion [10]. *Candida albicans* infections causing systemic candidiasis are becoming a social problem. This strain is prone to biofilm formation that is difficult to remove by mechanical or chemical cleaning [11]. Additionally, the model strain of the yeast *Saccharomyces cerevisiae*, despite its use in the food sector, may lead to pathological changes in respiratory, urinary, and digestive systems, especially in people with diabetes, where the source of excessive multiplication in the body may be the consumption of contaminated food [12]. The *Penicilium expansum* strain is also responsible for contamination in the food industry, particularly in fruit. It is expansive, as it has the ability to produce a very large number of spores in a short time [13].

Due to the wide application possibilities of the low-density polyethylene (LDPE)-based material being developed, this study was carried out with all of the above-described strains of bacteria and fungi. They are well-known sources of infections present in the medical sector, water, sanitary and ventilation systems, and the food industry.

Composites containing metal nanoparticles, especially silver, are used in many products, including food packaging. When applied as a component of the materials’ inner layer, they turned out to be an effective biocide against pathogenic microorganisms [14]. Nanometals, like their macro counterparts, are polycrystalline materials. Additionally, reducing the size of the particles increases the area of the active surface, increasing their chemical reactivity and strength [15]. Metals such as aluminum (Al), copper (Cu), iron (Fe), nickel (Ni), cobalt (Co), platinum (Pt), palladium (Pd), silver (Ag), and gold (Au) are most often miniaturized to a size of several nanometers. [16]. In the opinion of leading experts, the industrial use of nanomaterials is currently the most promising innovation for most key sectors of industry in the global economy [17]. It has been estimated that on average, three to four new nanoproducts are released on the market every week, which proves the potential application of nanomaterials [18]. Plastics containing components with nanoparticles show longer-lasting biocidal properties, compared to materials with a biocidal coating. This is because the entire material displays biocidal effects, and despite the surface wear of the material, the gradual release of particles is slower [19].

The most commonly used silver nanoparticles (nanoAg) show strong antibacterial activity against drug-resistant strains of Gram-positive and Gram-negative bacteria, such as *E. coli*, *S. aureus*, *Salmonella typhi*, and *Staphylococcus epidermidis*, as well as against strains of fungi, viruses, and parasites [20]. Compared to silver ions and silver complexes, silver nanoparticles have lower cytotoxicity or genotoxicity. Compared to antibiotics, nanosilver has a lower tendency to induce microbial resistance [21].

A composite of zinc oxide (ZnO) nanoparticles intercalated with montmorillonite used in HDPE materials was recognized as an inexpensive and non-toxic antibacterial and antifungal agent [22]. The effectiveness of ZnO is comparable to that of nanoparticles [23].

It has been known for decades that microbes and viruses are quickly inactivated by copper and its alloys. Numerous studies have shown that Cu has a stronger activity than other metals, such as Ag and Zn, in destroying bacteria, fungi, and viruses. Product surfaces containing copper are considered to be a non-toxic, cost-effective, and environmentally friendly method of preventing the spread of pathogens. The US Environmental Protection Agency (EPA) confirmed an average of 99% antimicrobial efficacy within 2 h for over 500 alloys containing ≥60% Cu [24]. In laboratory tests, biocidal efficacy has been demonstrated against pathogenic microorganisms such as the bacteria *S. aureus*, *E. coli*, and *Acinetobacter baumani* and the fungi *C. albicans*, *P. aeruginosa*, and *Kliebsiella pneumoniae* [25].

In addition to nanoparticles, naturally occurring compounds such as plant extracts (e.g., thymol, coumarin, green tea extract), agents of animal origin (chitin, chitosan, calcined waste shells as a source of CaO), and bacteriocins (natamycin, ampicillin) are used as biocides [11,26,27,28]. However, their effectiveness is short-lived, often weaker, and more expensive, and they also require higher dosing compared to nanometals. Typically, agents containing metal nanoparticles as active substances require dosing below 5 wt.% [29]. Moreover, inorganic biocides, such as metal and metal oxides, have an advantage over organic agents, which often cannot withstand the high-temperature conditions needed for processing polymeric materials [30].

The comprehensive use of nanoparticles in various industrial sectors, especially as an additive to packaging of food products, medical implants, and cosmetics, raises a controversy related to the risk of the migration and excessive accumulation of these particles in living organisms [31,32]. It has been proven that during their entire life cycle, nanocomposites may degrade due to exposure to environmental conditions, causing the release of deposited nanomaterials from the polymer matrix into the environment [33]. Hence, it is very important to ensure that metal nanoparticles do not migrate outside the material used. The phenomenon of migration is incorrectly considered as a dangerous factor only for compounds containing metals. Scientific research indicates the presence of heavy metals in living organisms from which some natural biocidal materials are obtained. These include mussels or oysters with high concentrations of metals such as Ba, Zn, Pb, Ni, Co, Cr, Sr, Cu, Mn, and Fe [34].

The thermodynamic stabilization of nanoparticles is achieved by adding blocking agents, e.g., polymers, saccharides, and oligosaccharides, which bind to the nanoparticle surface through covalent bonds or by chemical interaction. These blocking agents are also necessary to prevent nanoparticle aggregation [21,35]. It has been shown that the intensity of nanoAg migration depends on the environment in which the product containing them is found, and the process itself is more intense in an acidic environment [14]. According to the latest research studies described in the literature, it has been hypothesized that the use of iron oxide nanoparticles (Fe_2_O_3_) will facilitate the stabilization of nanoAg, nanoCu, and nanoZnO in a polymeric material and ensure their uniform dispersion without the use of any additional stabilizers [19].

Although it is assumed that iron nanoparticles themselves do not exhibit antibacterial and antifungal properties, they are used as a binding and stabilizing element for other nanoparticles, including Ag, Cu, Co, and Cr. Moreover, Fe compounds can be successfully used in processing, including steel processing, because they do not emit gases during degradation [19]. Nano-fillers are more and more often incorporated into polymeric materials not only to provide microbiological protection but also to improve the mechanical, barrier, or other properties of nanocomposite matrices used in consumer and industrial applications [33].

Often, the addition of a biocide containing nanoparticles does not have a positive effect on the biocidal properties of the polymer. This is related to problems involving the uniform dispersion of nanoparticles and their agglomeration ability in polymeric materials; hence, microscopic evaluation and elemental analysis on the surface of the samples were performed on the developed polymer material.

The aim of this study was to determine whether the combined action of metal nanoparticles (Ag, Cu, ZnO, Fe_2_O_3_) would ensure appropriate biocidal properties of LDPE materials against a wide range of microorganisms. According to the research hypothesis, appropriately selected concentrations of the applied metal nanoparticles allow for a high level of biocidal activity of polymers against pathogenic strains of bacteria and fungi, while simultaneously ensuring the safety of use due to the lack of the migration of particles to the environment. If the results confirm the hypothesis, they will permit the use of these types of materials in industry for the production of pipes and fittings used in water supplies and sanitary and/or ventilation systems by confirming their safety of use and protection against harmful microorganisms. The use of the developed materials will extend the efficient functioning of the installations under construction. The material can also be used in the medical sector for the production of fittings and equipment, as well as in the food sector as a packaging material.

## 2. Materials and Methods

### 2.1. Biocide

The biocide was provided by ZPTS Ingremio-Peszel (Poland), a company that commercially deals with the development and production of polymers and finished polymer products, functionalized with nanocomponents to obtain microbiological protection. According to the specification, a concentrate of nanomaterials in the odorless form of yellow-brown granules was prepared, containing nanoparticles with a diameter of 5–15 nm (for 95% of particles) containing approximately 150 ppm nanoAg, 100 ppm nanoCu, 500 ppm nanoZnO, and 150 ppm nanoFe_2_O_3_.

### 2.2. Sample Preparation

Samples of LDPE Malen E Fabs 23-D022 granules (LyondellBasell, Rotterdam, The Netherlands) and composites containing the biocide at concentrations of 0.5, 1, 2, 3, 4, and 5 wt.%, respectively, were prepared. For this purpose, the composites were extruded using a BTSK 20/40D co-rotating twin screw extruder (Bühler, Alzenau, Germany). The extrudate was dried on a conveyor belt in a stream of dry air and then granulated.

Films were extruded from the granulate using a laboratory technological line consisting of a Plasti-Corder PLV 151 single-screw extruder (Brabender, Duisburg, Germany) with a slot die with a working width of 170 mm and a gap height of 0.35 mm and a water-cooled three-roll honing system with a diameter of 110 mm rolls. A 25D screw with an 8D mixing tip and a 3:1 compression ratio was used. Additionally, the station was equipped with the apparatus necessary to measure the temperature of the heating zones of the plasticizing system and the die. 

After extrusion, the thickness of the film was about 0.1 mm. Samples with a size of 50 × 50 mm (±2 mm) were cut from each type of film.

### 2.3. Microorganisms

The antibacterial activity study of the polymer material samples was carried out with the following strains: *E. coli* (ATCC 8739), *S. aureus* (ATCC 6538P), *P. aeruginosa* (ATCC 13388), *L. pneumophila* (WDCM 00107, ATCC 33152), and *S. enterica* subsp. *enterica* (ATCC 51741). Prior to testing, the *L. pneumophila* strain was cultured for 72 h at 35 °C in Petri dishes containing BCYE (Buffered Charcoal Yeast Extract medium (BD BTL, Greeneville, TN, USA)). The remaining strains were cultured according to ISO 22196:2011 [5] for 24 h at 35 °C (±1 °C) in NA (Nutrient Agar (Difco, Baltimore, MD, USA)) and then transplanted onto fresh media and re-cultured for 20 h.

The study of the fungistatic properties of the samples of the polymer material was carried out with the use of the following strains: *A. brasiliensis* (ATCC 9642), *C. albicans* (ATCC 10231), *S. cerevisiae* (WDCM 00058, ATCC 9763), and *P. expansum* (KKP 774). Prior to testing, the strains were cultured for 5 days at 29 °C (±1 °C) in Petri dishes containing PDA (Potato Dextrose Agar (Difco, Baltimore, MD, USA)).

### 2.4. Examination of Bactericidal Properties

Bactericidal properties tests were carried out in accordance with ISO 22196:2011 (Measurement of Antibacterial Activity) [5], which is an international standard compatible with JIS Z 2801:2000 [6]. In addition to the standard strains of *E. coli* (ATCC 8739) and *S. aureus* (ATCC 6538P), additional reference strains are described in p. 2.3.

For each of the strains, the study was conducted on three samples from each test material (containing the biocide) and on six samples of the control material (without the biocide). Half of the control samples were used to measure viable cells immediately after inoculation and half were used to measure viable cells after 24 h of incubation. The samples did not require sterilization prior to testing. The inoculum volume was 0.4 mL. The covering layer was a polyethylene foil with dimensions of 40 × 40 mm (±1 mm) and a thickness of 0.06 mm. Petri dishes containing inoculated test samples (including half of the control samples) were incubated at 35 °C (±1 °C) and 90% relative humidity (RH) (±5%) for 24 h (±1 h).

For each tested sample, the number of live bacteria recovered was determined in accordance with the guidelines of ISO 22196: 2011 [5]. According to the standard guidelines, the concentration of individual bacteria was 6 × 10^5^ CFU/mL. The antimicrobial activity was then expressed as the decimal logarithm CFU/cm^2^ reduction relative to the control sample.

### 2.5. Study of Fungistatic Properties

The fungistatic properties tests were carried out in accordance with the methodology outlined in ISO 846:2019 (Plastics. Evaluation of the activity of microorganisms), using method B: Determination of the fungistatic effect, on the standard fungal strains described in point 2.3. According to the standard guidelines, the concentration of individual fungal spores was approximately 10^6^ spores/mL. In accordance with the guidelines of the standard, the samples were divided into test batches: (i) batch 0—control samples (2 pieces each), stored in standardized climatic conditions of conditioning and testing in accordance with EN ISO 291:2010 (23 ± 1 °C, 50 ± 5% RH); (ii) batch S—sterile samples (2 pieces each), stored in the same conditions as batch I; and (iii) batch I—test samples (5 for each sub-batch: I_a_—without biocide and I_b_—with biocide) inoculated with microorganisms and incubated. Microorganism suspensions were prepared in accordance with the guidelines of the standard. Incubation was at 29 ± 1 °C and 90% RH (±5%) for 28 days. The visual assessment was performed on the basis of photos taken with an automatic SCAN 1200 colony counter (Interscience, Saint-Nom-la-Bretèche, France). An Olympus SZX 12 stereoscopic microscope (Olympus, Waltham, MA, USA) was used for microscopic observations at 42× magnification of the sample image, using an Artcam 300 MI camera (Artray, Taipei, Japan).

### 2.6. Assessment of Dispersion

The evaluation of biocide dispersion in the LDPE plastic was carried out using a scanning electron microscope, SEM (Hitachi SU8010, Tokyo, Japan). Fragments of 20 × 20 mm were cut from the samples and sputtered with a 1 nm gold coating using a gold sputtering machine (Cressington Sputter Coater 108 auto, Watford, UK) with a sputter thickness measurement module (Cressington Thickness Monitor mtm10, Watford, UK). The photos were taken at 1000× magnification and a voltage of 30 kV. Elemental analysis of the film samples surface was performed using energy dispersive X-ray analysis (EDX) using the SEM-EDX attachment (Thermo Scientific Ultra Dry, Pittsburgh, PA, USA). The EDX spectrum of the sample surface allows for a semi-quantitative analysis of the elemental composition to a depth of about 1 µm [36]. During the EDX analysis, the samples were subjected to a voltage of 30 kV and intensity of 15 μA. Elemental analysis of the surface took 30 s at 100× magnification and 15 mm working distance.

### 2.7. Migration Studies

Global migration tests were performed in an independent accredited laboratory (J.S. Hamilton Poland Sp.z o.o., Gdynia, Poland). The tests were carried out in accordance with the EN 1186:2005 standard using four model fluids: 10% ethanol, distilled water, 3% acetic acid, and isooctane. For isooctane, the contact conditions were: 2 days at 20 °C (according to EN 1186-14:2005) [37]; for the remaining model fluids, contact conditions were 10 days at 40 °C (according to EN 1186-3:2005) [38]. The contact area/volume of the model fluid was 2.11 dm^2^/100 mL. According to the guidelines, the acceptability criterion is ≤10 mg/dm^2^.

### 2.8. Statistical Analysis

Figures are presented as mean ± standard deviation. The obtained results were analyzed using the Past 321 program (Softpedia, Past 321, Bucharest, Romania, 2018). Significant differences were determined on the basis of one-way analysis of variance (ANOVA) using Tukey’s test for *p* < 0.05.

## 3. Results

### 3.1. Examination of Bactericidal Properties

According to research on the methodology used, the antibacterial properties of polymer materials are determined by measuring the reduction of antibacterial activity (R), i.e., on the basis of the difference in the logarithm of the number of living cells found on the material containing the biocide and the control material after inoculation with bacteria and incubation. The antimicrobial product is assumed to have antimicrobial efficacy when R ≥ 2.0. The number of viable bacterial cells after sample incubation is presented in Table 1. The results met the validation conditions according to ISO 22196:2011 [5].

**Figure 1:**
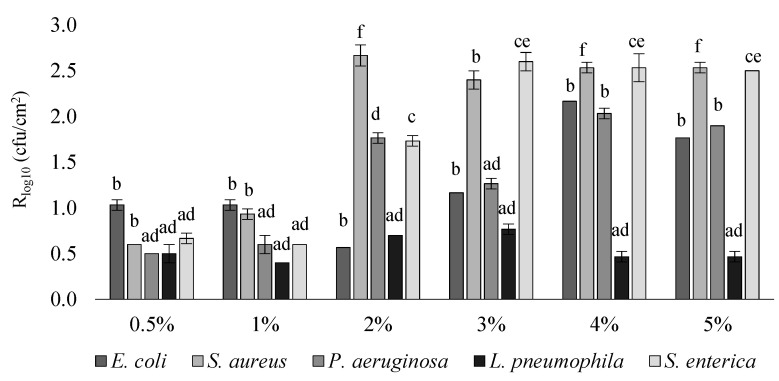
Antimicrobial activity (R) values of samples containing the biocide at a concentration of 0.5–5% (x-axis) relative to the control samples (without biocide); all R values are statistically significant in relation to the control samples. Statistically significant differences between the variants in the graph are marked with different letters, for *p* < 0.05.

Based on the evaluation of bacteria (Table 1), we conclude that after 24 h incubation, the lowest live count of *E. coli* was observed on samples containing 3–5% biocide. The fewest *S. aureus* cells survived on the samples containing 2% of the biocide. Even at a concentration of 0.5%, the biocide significantly reduced the abundance of *P. aeruginosa* and *S. enterica*. There were no statistically significant differences in the number of *L. pneumophila* strains. Subsequently, based on the number of bacteria (Table 1), R was calculated for each variant. The results are shown in Figure 1.

For all percentagesof biocide contents, R ≥ 0.4 was observed. A significant increase in R was observed for samples containing ≥2% biocide, especially against strains of *S. aureus* < *S. enterica* < *P. aeruginosa*. With regard to the *S. aureus* strain, the highest values of R > 2.5 were observed for the biocide content in the range of 2–5%. Comparable R values were recorded for the *S. enterica* strain for samples containing 3–5% biocide, although a very high R value of approximately 2.0 was also observed at 2%, corresponding to a reduction of CFU/cm^2^ by approximately 99%. An R value of approximately 2.0 for *P. aeruginosa* was only observed at 4–5% biocide content. The increase in activity against *E. coli* was observed with the presence of ≥4% biocide, but despite the observed significant decrease in the number of bacteria (Table 1), the difference in R was not statistically significant. There were no statistically significant differences in R against *L. pneumophila* regardless of the percentage of biocide content (Figure 1).

### 3.2. Study of Fungistatic Properties

**Figure 2:**
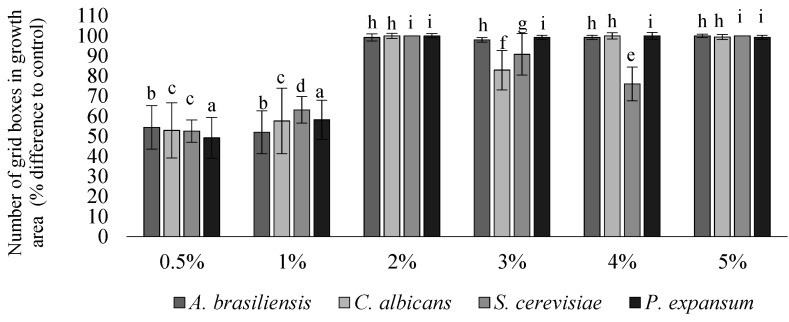
Assessment of fungal growth on samples with 0.5–5% biocide content (x-axis) using a growth area grid according to ISO 846:2019 [7] (percentage difference fromcontrol). All values are statistically significant in relation to the control samples. Statistically significant differences between the variants in the diagram are marked with different letters, for *p* < 0.05.

The fungistatic properties of the developed LDPE materials were determined using Method B, according to ISO 846:2019 [7], with the use of *A. brasiliensis*, *C. albicans*, *S. cerevisiae*, and *P. expansum* strains. Any inhibition of fungal growth, both on the surface of the polymeric material sample and around it (inhibition zone) indicates the fungistatic activity of the polymeric material. As expected, no fungal growth was observed on the samples of batch S (sterile samples), which confirms the lack of sample contamination during incubation. For ease of interpretation, samples from batch I_a_ were labeled control, and samples from batch I_b_ were labeled according to their percentage of biocide content, analogous to the bacterial tests. The visual assessment of the size of the surface of the specimen overgrown with fungi was carried out using a grid with dimensions of 50 × 50 mm (±0.5 mm), divided into 100 equal squares. According to the guidelines, the outer 36 squares were not taken into account. The results of the number of squares covered with fungal growth on the samples are presented in Figure 2.

The greater the difference in the percentage of the overgrown area of samples with biocide compared to the control, the greater the fungistatic effect (Figure 2). In the samples containing 0.5% or 1% biocide, inhibition of growth was observed in the range of 49–63% compared to the control samples (without biocide), of which the most intensive growth was observed for sporulating fungi (*A. brasiliensis* and *P. expansum*). In samples containing 2–5% of biocide, a significant reduction in growth or even complete inhibition (76–100%) of all four fungal strains was observed. The growth of yeasts (*C. albicans* and *S. cerevisiae*) was least inhibited. In samples containing 4% and 5% biocide, no statistically significant differences were observed in the growth of all the fungal strains used, except for *S. cerevisiae*, whose growth was significantly reduced under the influence of 5% biocide (Figure 2).

According to the scale adopted in the ISO 846:2019 standard, the appropriate percentage of the covered area was assigned the following grades: (0–1) no growth; (2) an increase of up to 25%; (3) an increase of 26% to 50%; (4) an increase of more than 50%. Confirmatory microscopic analysis was performed for samples where no fungal growth was observed by visual inspection. During microscopic observations, the samples were assessed according to a scale: (0) no visible growth; (1a) growth covering up to 25% of the area; (1b) growth covering up to 50% of the area; and (1c) growth covering more than 50% of the area. The results are included in Table 2.

**Figure 3:**
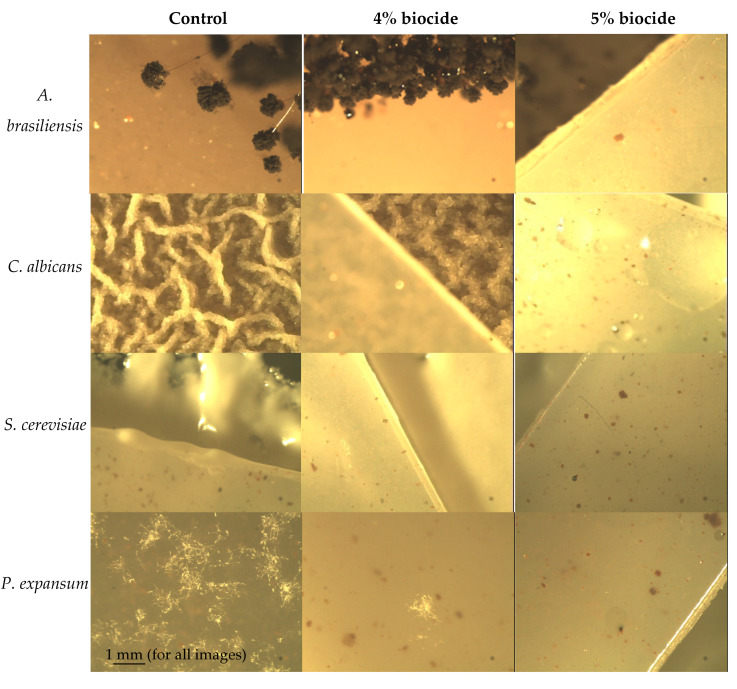
Microscopic evaluation of fungal growth on samples at 42× magnification of the sample image (selected variants).

The results of the microscopic analysis did not significantly affect the final result of the determination of the fungistatic effect. In the microscopic evaluation, only an intense edge growth was observed, which according to the standard was not taken into account in the evaluation. A fungistatic effect was observed for all samples with the biocide. When the score 0 is assigned to the samples according to the described scale, we can say there is a fungicidal effect because no growth of fungi was observed on the sample, both in visual and microscopic evaluation. Surprisingly, the observed fungicidal effect against *S. cerevisiae* for samples with 2% biocide rated 0, while a weaker fungistatic effect (rating 2) was observed for samples containing 3% and 4% biocide (Table 2). Indeed, for samples with 3% and 4% biocide, a slightly higher average percentage of the covered area was observed, but the analysis of the results showed very high values of the standard deviation for these variants (Figure 2). There were no statistically significant differences between the samples containing 2% and 5% biocide. The conducted analyses indicate the great importance of statistical analysis in relation to the obtained results. Summarizing the obtained results, a strong inhibition of growth of all fungal strains used was recorded for samples containing 2% or more of biocide. To illustrate the fungistatic effect of the samples, Figure 3 presents selected photographs from the microscopic evaluation.

### 3.3. Assessment of Biocide Dispersion

**Figure 4:**
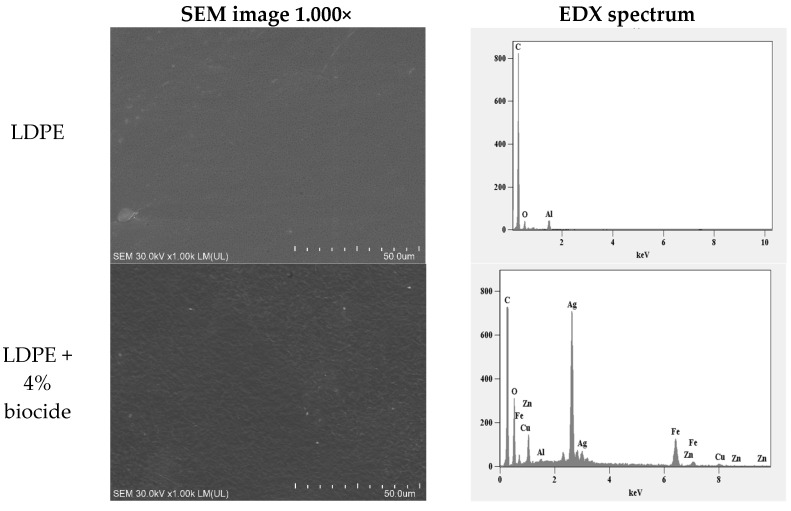
Scanning electron microscope (SEM) images showing the LDPE control and LDPE composite containing 4% biocide at 1000× magnification and their corresponding EDX spectra.

The polymer material with the best biocidal properties against bacteria and fungi with the lowest possible biocide content was subjected to further analyses. For comparative purposes, the surface structure was observed using SEM for a material containing 4% biocide compared to a material without biocide (Figure 4). In order to assess the dispersion of metal nanoparticles in the material, elemental analysis was performed using SEM-EDX (Figure 4).

SEM images showed differences in the surface structure of samples with and without the biocide. Luminous points were observed in the photos, probably corresponding to the presence of nanoparticles (Figure 4). EDX analysis confirmed these results. For control samples, the main presence of the following elements was indicated: C (79.68 wt.%), O (16.76 wt.%), and a small amount of Al (3.56 wt.%). For samples with the biocide, similar to the control, the most abundant elements were indicated as: C (44.99 wt.%), O (44.69 wt.%), and a small amount of Al (0.33 wt.%). Contrary to the control samples, Fe (5.90 wt.%), Cu (0.54 wt.%), Zn (0.09 wt.%), and Ag (3.46 wt.%) were among the least added metals (Figure 4). The glowing points observed in the SEM analysis were mostly arranged singly, although small places of compaction were also observed, but they did not touch each other (Figure 4). The analysis confirmed we had achieved the desired uniform dispersion of metal nanoparticles in the LDPE sample. The small Al peak visible in the EDX spectra is most likely an artifact of electrons knocked out of the SEM sample stage.

### 3.4. Migration Studies

Similar to the dispersion evaluation, the material with the lowest possible biocide content but best biocidal properties against bacteria and fungi qualified for the migration tests (Table 3).

The migration tests for the LDPE sample containing 4% biocide showed results significantly below the maximum acceptability criterion (10 mg/dm^2^), i.e., below 0.5 mg/dm^2^ (in 10% ethanol, distilled water, and 3% acetic acid) and below 0.6 mg/dm^2^ (in isooctane) (Table 3). The results confirm the negligible migration of particles after incubation in food stimulants. This is a beneficial effect enabling the potential use of the developed materials in various applications, including the medical or packaging sector.

## 4. Discussion

In order to confirm the hypothesis that the combined action of metal nanoparticles (Ag, Cu, ZnO, Fe_2_O_3_) will ensure the appropriate biocidal properties of LDPE against microorganisms, the bactericidal and fungistatic properties of samples containing from 0.5% to 5% of biocide were tested. According to the literature review, more than 2880 scientific publications have been published since 2007, including 75% in the field of material science and chemistry, concerning antibacterial coatings, mainly based on nanoAg [39]. Bazant et al. [40] investigated the effect of a combination of Ag–ZnO as an additive to polypropylene (PP) at a concentration of up to 5 wt.%. They found high antibacterial efficacy against *E. coli* and *S. aureus* in each of the percentages by weight used. No reports were found in literature on the simultaneous use of Ag, Cu, ZnO, and Fe_2_O_3_ nanoparticles.

Very restrictive tests of bactericidal properties carried out in accordance with the international standard showed effectiveness against difficult-to-control strains, with the highest value of reduction of antibacterial activity recorded against *S. aureus* < *S. enterica* < *P. aeruginosa*. Samples containing the biocide turned out to be the least effective against *L. pneumophila*, and against *E. coli*, no statistically significant differences were found between the various concentrations of the biocide (0.5–5%). Due to the observed increase in the biocide efficiency at the content of 4% and 5% against *P. aeruginosa*,the optimal concentration seems to be the production of the LDPE composite with 4% biocide. Research by Sawai et al. [41] with the presence of natural origin substances (shell powder), contrary to ours, showed greater biocidal effectiveness of the agents used against *E. coli* than *S. aureus*, *S. typhimurium*, and *B. subtilis*.

Apart from the *E. coli* and *S. aureus* strains compliant with the [5] standard, other pathogenic reference strains of bacteria were used: *P. aeruginosa*, *L. pneumophila*, and *S. enterica*. The obtained results prove the broad spectrum of activity of the applied biocide against microorganisms that are difficult to fight. The selection of the most appropriate biocide concentration was determined by the tests in the presence of *P. aeruginosa* and not by the standard strains tested in the norm (*E. coli* and *S. aureus*).

For certain nanometals, mainly nanoZn and nanoCu but excluding nanoAg, percentage content is undoubtedly the most influential factor regarding the microbiological effectiveness of the developed material. Shafaghi et al. [42] observed that, under normal atmospheric conditions, exposure of *Bacillus subtilis* endospores to Cu leads to endospore morphology destruction and inactivation within a few hours. Cu and Cu alloys proved to be effective in deactivating COVID-19/SARS-CoV-2 [43]. Lomate et al. [44] modified LDPE by adding nanoCu in amounts of 0.5 wt.% up to 30 wt.% and found very high antibacterial activity of the material against *E. coli* and *S. aureus*, directly proportional to the biocide concentration used.

Tests carried out with fungi, in accordance to the international standard [7] by definition relate to the study of fungistatic properties. However, interpretation of the obtained results, in which no growth of fungi on the sample was observed in both visual and microscopic evaluation, allows us to draw conclusions on the fungicidal properties. A strong inhibition of growth of all fungal strains used was recorded for samples containing 2% or more of the biocide. Therefore, the content of the biocide is half that of the bacteria.

As with the studies with bacteria, in addition to the standard strains used, pathogenic reference strains of fungi were included. Here, too, the results obtained confirm the broad spectrum of activity for the biocide used. Additionally, yeasts (*C. albicans* and *S. cerevisiae*) turned out to be more difficult to control than spore-forming fungi (*A. brasiliensis* and *P. expansum*). Additionally, Li et al. [45] indicated the potential of nanoAg to fight *C. albicans* and inhibit its adhesion and biofilm production on polymeric materials, but only at 5% biocide concentration. The demonstrated broad spectrum of biocidal activity against the tested microorganisms is very important because, as the research shows, the effectiveness against strains listed in the standards does not always translate into antimicrobial effectiveness under real conditions. The different effects of the same substance on bacterial strains were shown, among others, by Nanda et al. [46] evaluating the effectiveness of nanoAg in terms of its antibacterial activity against various pathogenic organisms. Scientists found antibacterial activity against methicillin-resistant *S. aureus* (MRSA), *Staphylococcus epidermidis* (MRSE), and *Streptococcus pyogenes*, while only moderate antibacterial activity was observed with *Salmonella typhi* and *Klebsiella pneumoniae*.

Materials with 4 wt.% of biocide containing metal nanoparticles proved to be the most effective. Meanwhile, for substances of natural origin, the percentage of dosing in order to ensure biocidal properties against *E. coli* and *S. aureus* in accordance with the standards applied is much higher and may even be as high as 9–11% [28]. Additionally, for ZnO nanoparticles, it was found that a slightly higher (5%) concentration was most optimal to be effective in combating *E. coli*, *S. aureus*, and *A. niger* [22].

The high efficiency of the biocide used (in this study) as a component of the polymeric material was influenced by the appropriate dispersion of the agent. This was confirmed by elemental analysis (EDX), microscopic analysis (SEM), and the patented production method reserved for international patent application PCT/PL2021/000012 (unpublished data). The proprietary method is based on adding a marker to the active compounds of the biocide that emits visible light when exposed to UV radiation. The emitted light is recorded and measured with a fluorimeter. The described method of dispersion evaluation was used to test materials of the composition produced under identical production conditions. Ziąbka et al. [47] probably did not observe antibacterial activity against *E. coli* and *S. aureus* in the presence of HDPE with 0.5% and 1% addition of nanoAg biocide due to the aggregation and non-uniform agglomeration of nanoAg in the matrix. Generally, there are more reports on problems with aggregation and agglomeration of nanoAg in a polymeric material than other nanometals. Chen et al. [48] confirmed microscopically that there were no problems with the uniform distribution of nanoCu in PMMA, which consequentially showed a very high (approximately 99%) biocidal activity against *E. coli.*

Undoubtedly, nanoFe_2_O_3_ contained in the biocide played a role in the stabilization of Ag, Cu, and ZnO nanoparticles. Although no reports on the stabilization of Fe nanoparticles in polymers were found in the literature, nanoFe proved to be effective in stabilizing the residues after processing chromium ore in groundwater [49]. At the same time, although Fe ions are considered to be neutral towards microorganisms, there is insufficient knowledge on this subject. Meanwhile, Wang et al. [50] observed a complete inhibition of the growth of *Photobacterium phosphoreum* in the presence of Fe^3+^ at a concentration of 0.01 mg/L.

Migration studies confirmed the effective immobilization of nanometal particles inplastic. The obtained results indicate that the tested material not only meets the requirements for use as a construction material, but it can also be approved for contact with food in accordance with the European Union Regulation on plastic materials and products intended to come into contact with food [51]. The results of this study, compared to the conducted literature analysis, indicate the innovative nature of the tested material with high implementation potential. Often, during the development of biocidal materials containing metal nanoparticles, such as Ag and others, high biocidal effectiveness was associated with strong migration, which discredited the obtained material from the market launch. In the developed material, nanoparticles are immobilized in the LDPE surface layer against adhering microorganisms, without migrating to the environment. Cushen et al. [52] used distilled water to measure migration for a PE composite containing 0.5% of a biocide with nanoAg. They obtained permissible values of ≤10 mg/dm^2^. In the presented study, the biocide used contained nanometal particles with sizes of 5–15 nm (for 95% of nanoparticles). Research by Bott et al. [53] showed that polymeric material additives with larger particle sizes have higher migration. Ozaki et al. [54] found the highest migration of Ag and Zn particles in 4% acetic acid, although significant values were also obtained after incubation of the samples in water and 20% ethanol. Researchers found a greater proportion of Ag in the form of nanoparticles in acetic acid and in its ionic form in the remaining media. Similar observations were made by Hauri and Niece [55] studying the migration of Ag nanoparticles from commercially available plastic containers. In our research, the type of medium did not significantly affect the differences in particle migration. Guaranteeing no migration of nanoparticles is extremely important, especially since there are reports on the negative impact of metals, especially silver, on human health. When used in excess, they can induce oxidative stress and cause a disturbance of the cellular redox system and the formation of reactive oxygen species [56]. However, according to researchers, nanoZnO reduces the bioavailability of lead and cadmium and increases the content of iron and copper in leafy greens [57].

The obtained LDPE material with 4 wt.% biocide content, showing no migration of metal nanoparticles, and with very high microbiological activity against pathogenic strains of bacteria and fungi is a very important result from the point of view of the application properties of the tested materials. Its inclusion in the research on pathogenic microorganisms contributing to hospital, installation (water, sewage, sanitary, and/or ventilation) infections, and food infections allows for multidirectional practical applications, e.g., as an inner layer of pipes or fittings. Before the final application of the tested material, it is reasonable to test the migration of nanoparticles from the polymer material in conditions similar to real life ones, especially temperature conditions. High temperatures accelerate the migration of substances from polymeric materials [58].

## 5. Conclusions

The obtained results show that the combined use of Ag, Cu, ZnO, and Fe_2_O_3_ nanoparticles results in very high antimicrobial activity of the polymers with a low (4%) biocide dosing. Metal nanoparticles immobilized in the LDPE surface layer significantly reduced the growth of microorganisms adhering to the material. It is an innovative, relatively cheap, and, above all, safe solution for the use of biocidal materials, effective against a wide range of bacteria and fungi.

## Figures and Tables

**Table 1:** Number of viable bacterial cells after incubation CFU/cm^2^ (mean ± standard deviation in brackets). Statistically significant differences in the columns are marked with different letters. Control: sample without biocide; 0.5–5%: samples with appropriate biocide content; *: statistically significant differences from control for *p* < 0.05.

Strain:	*E. coli*	*S. aureus*	*P. aeruginosa*	*L. pneumophila*	*S. enterica*
Control	1.4 × 10^3^ (120) b	3.7 × 10^2^ (10) b	7.9 × 10^3^ (680) d	2.4 × 10^3^ (150) a	4.8 × 10^3^ (670) c
0.5%	1.4 × 10^2^ (12) ab	9.8 × 10^1^ (10) ab	2.6 × 10^3^ (150) bc *	7.8 × 10^2^ (110) a	1.1 × 10^3^ (150) b *
1%	1.4 × 10^2^ (15) ab	4.6 × 10^1^ (7) ab	2.3 × 10^3^ (58) bc *	1.0 × 10^3^ (85) a	1.3 × 10^3^ (100) b *
2%	3.8 × 10^2^ (10) ab	0.8 (0) a *	1.4 × 10^2^ (10) bc *	5.3 × 10^2^ (6) a	9.2 × 10^2^ (8) ab *
3%	1.0 × 10^2^ (6) a *	1.6 (0) ab	4.4 × 10^2^ (15) bc *	4.5 × 10^2^ (12) a	1.3 × 10^1^ (3) a *
4%	1.2 × 10^1^ (1) a *	1.2 (0) ab	7.4 × 10^1^ (5) b *	8.6 × 10^2^ (97) a	1.5 × 10^1^ (5) a *
5%	2.8 × 10^1^ (0) a *	1.2 (0) ab	1.1 (0) a *	8.1 × 10^2^ (85) a	1.6 × 10^1^ (0) a *

**Table 2:** Test results for the determination of fungistatic properties of polymer samples in accordance with ISO 846:2019.

	*A. brasiliensis*	*C. albicans*	*S. cerevisiae*	*P. expansum*
Ctr	3	3	3	4
0.5%	2	2	2	2
1%	2	2	2	2
2%	0	0	0	0
3%	0	2	2	0
4%	0	0	2	0
5%	0	0	0	0

**Abbreviations:** Ctr: control sample (without biocide); 0.5–5%: corresponding concentration of the biocide; 0: no visible growth under the microscope; 1: growth invisible to the naked eye but clearly visible under the microscope, including: 1a: ≤25%, 1b: ≤50%, and 1c: >50% of the sample surface; 2: increase of ≤25% of the tested area; 3: increase of ≤50% of the tested area; 4: increase >50% of the tested area.

**Table 3:** Global migration test results for LDPE containing 4% biocide in accordance with EN 1186-1:2015 (acceptability criterion ≤ 10 mg/dm^2^).

Model Fluid	Contact Conditions	Result (mg/dm^2^)
10% ethanol	10 days at 40 °C	0.2 ± 0.10
Distilled water	10 days at 40 °C	0.4 ± 0.06
3% acetic acid	10 days at 40 °C	0.3 ± 0.06
Isooctane	2 days at 20 °C	0.6 ± 0.50

## Data Availability

All data is contained within the article.

## References

[1] Pemmada R., Zhu X., Dash M., Zhou Y., Ramakrishna S., Peng X. (2020). Science-based strategies of antiviral coatings with viricidal properties for the COVID-19 like pandemics. Materials.

[2] Gabriel G.J., Som A., Madkour A.E., Eren T., Tew G.N. (2007). Infectious disease: Connecting innate immunity to biocidal polymers. Mater. Sci. Eng. R. Rep..

[3] Kugel A., Stafslien S., Chisholm B.J. (2011). Antimicrobial coatings produced by “tethering” biocides to the coating matrix: A comprehensive review. Prog. Org. Coat..

[4] Laverty A.L., Primpke S., Lorenz C., Gerdts G., Dobbs F.C. (2020). Bacterial biofilms colonizing plastics in estuarine waters, with an emphasis on Vibrio spp. and their antibacterial resistance. PLoS ONE.

[5] ISO 22196 (2011). Measurement of Antibacterial Activity on Plastics and Other Non-Porous Surfaces.

[6] JISZ 2801 (2010). Antibacterial Products–Test for Antibacterial Activity and Efficacy.

[7] ISO 846 (2019). Plastics—Evaluation of the Action of Microorganisms.

[8] Declerck P. (2010). Biofilms: The environmental playground of Legionella pneumophila. Environ. Microbiol..

[9] Vinayaka A.C., Ngo T.A., Kant K., Engelsmann P., Dave V.P., Shahbazi M.A. (2019). Rapid detection of Salmonella enterica in food samples by a novel approach with combination of sample concentration and direct PCR. Biosens. Bioelectr..

[10] Amanati A., Lotfi M., Masoudi M.S., Jafarian H., Ghasemi F., Bozorgi H., Badiee P. (2020). Cerebral and pulmonary aspergillosis, treatment and diagnostic challenges of mixed breakthrough invasive fungal infections: Case report study. BMC Infect. Dis..

[11] Chen H., Han Q., Zhou X., Zhang K., Wang S., Xu H.H. (2017). Heat-polymerized resin containing dimethylaminododecyl methacrylate inhibits Candida albicans biofilm. Materials.

[12] Romanio M.R., Coraine L.A., Maielo V.P., Abramczyc M.L., de Souza R.L., Oliveira N.F. (2017). Saccharomyces cerevisiae fungemia in a pediatric patient after treatment with probiotics. Rev. Paul. Pediatr..

[13] He L., Liu Y., Mustapha A., Lin M. (2011). Antifungal activity of zinc oxide nanoparticles against Botrytis cinerea and Penicillium expansum. Microbiol. Res..

[14] Mackevica A., Olsson M.E., Hansen S.F. (2016). Silver nanoparticle release from commercially available plastic food containers into food simulants. J. Nanoparticle Res..

[15] Foltynowicz Z., Czajka B., Maranda A., Wachowski L. (2017). Aspects of nanomaterials for civil and military applications Part 1. The origin, characterization and methods of obtaining. High Energy Mater..

[16] Zarko V.E., Gromov A.A. (2016). Energetic Nanomaterials: Synthesis, Characterization and Application.

[17] Golubev S.S., Sekerin V.D., Gorokhova A.E., Gayduk N.V. (2018). Nanotechnology market research: Development and prospects. Rev. Espac..

[18] New Nanotech Products Hitting the Market at the Rate of 3–4 per Week. https://phys.org/news/2008-04-nanotech-products-week.html.

[19] Zhang E., Zhao X., Hu J., Wang R., Fu S., Qin G. (2021). Antibacterial metals and alloys for potential biomedical implants. Bioact. Mater..

[20] Ingle A., Gade A., Pierrat S., Sonnichsen C., Rai M. (2008). Mycosynthesis of silver nanoparticles using the fungus Fusarium acuminatum and its activity against some human pathogenic bacteria. Curr. Nanosci..

[21] De Lima R., Seabra A.B., Durán N. (2012). Silver nanoparticles: A brief review of cytotoxicity and genotoxicity of chemically and biogenically synthesized nanoparticles. J. Appl. Toxicol..

[22] Roy A., Joshi M., Butola B.S. (2019). Preparation and antimicrobial assessment of zinc-montmorillonite intercalates based HDPE nanocomposites: A cost-effective and safe bioactive plastic. J. Clean. Prod..

[23] Deryabin D.G., Aleshina E.S., Vasilchenko A.S., Deryabina T.D., Efremova L.V., Karimov I.F., Korolevskaya L.B. (2013). Investigation of copper nanoparticles antibacterial mechanisms tested by luminescent Escherichia coli strains. Nanotechnol. Russ..

[24] Mostaghimi J., Pershin L., Salimijazi H., Nejad M., Ringuette M. (2021). Thermal spray copper alloy coatings as potent biocidal and virucidal surfaces. J. Therm. Spray Technol..

[25] Inkinen J., Mäkinen R., Keinänen-Toivola M.M., Nordström K., Ahonen M. (2017). Copper as an antibacterial material in different facilities. Lett. Appl. Microbiol..

[26] Ha J.U., Kim Y.M., Lee D.S. (2001). Multilayered antimicrobial polyethylene films applied to the packaging of ground beef. Pack. Technol. Sci. Int. J..

[27] Richert A. (2017). Structural and barrier properties of polylactide films with bacteriocins after biodegradation in a compost extract. Przem. Chem..

[28] Kao C.Y., Huang Y.C., Chiu S.Y., Kuo K.L., Hwang P.A. (2018). Bacteriostatic effect of a calcined waste clamshell-activated plastic film for food packaging. Materials.

[29] Królikowski W., Rosłaniec Z. (2004). Polymer nanocomposites. Composites.

[30] Othman S.H. (2014). Bio-nanocomposite materials for food packaging applications: Types of biopolymer and nano-sized filler. Agricult. Agricult. Sci. Proced..

[31] Gallocchio F., Cibin V., Biancotto G., Roccato A., Muzzolon O., Carmen L. (2016). Testing nano-silver food packaging to evaluate silver migration and food spoilage bacteria on chicken meat. Food Addit. Contam. Part A.

[32] Qian S., Ji H., Wu X., Li N., Yang Y., Bu J. (2018). Detection and quantification analysis of chemical migrants in plastic food contact products. PLoS ONE.

[33] Han C., Zhao A., Varughese E., Sahle-Demessie E. (2018). Evaluating weathering of food packaging polyethylene-nano-clay composites: Release of nanoparticles and their impacts. NanoImpact.

[34] Al-Jaberi M.H. (2015). Heavy metal concentrations in the bivalve Corbicula fluminalis shells from Shatt Al-Arab River. Int. J. Marin. Sci..

[35] Durán N., Marcato P.D., Conti R.D., Alves O.L., Costa F., Brocchi M. (2010). Potential use of silver nanoparticles on pathogenic bacteria, their toxicity and possible mechanisms of action. J. Braz. Chem. Soc..

[36] Hockaday W.C., Grannas A.M., Kim S., Hatcher P.G. (2007). The transformation and mobility of charcoal in a fire-impacted watershed. Geochim. Cosmochim. Acta.

[37] (2005). EN1186-14 Materials and Articles Intended to Come into Contact with Food Products—Plastics—Part 14: Test Methods for Global Migration of Plastics Intended for Contact with Fatty Food Products in Substitution Tests Using Isooctane and 95 Percent Ethanol as Substitution Media.

[38] (2005). EN 1186-3 Materials and Articles Intended to Come into Contact with Foodstuffs—Plastics—Part 3: Test Methods for Global Migration to Aqueous Simulants by Total Immersion.

[39] Hamerliński J., Niciński K. (2020). Antimicrobial coatings-a review of current state of technology. Act. Poligraph..

[40] Bazant P., Sedlacek T., Kuritka I., Podlipny D., Holcapkova P. (2018). Synthesis and effect of hierarchically structured Ag-ZnO hybrid on the surface antibacterial activity of a propylene-based elastomer blends. Materials.

[41] Sawai J., Shiga H., Kojima H. (2001). Kinetic analysis of the bactericidal action of heated scallop-shell powder. Int. J. Food Microbiol..

[42] Shafaghi R., Mostaghimi J., Pershin V., Ringuette M. (2017). Sporicidal efficacy of thermal-sprayed copper alloy coating. Can. J. Microbiol..

[43] Cortes A.A., Zuñiga J.M. (2020). The use of copper to help prevent transmission of SARS-coronavirus and influenza viruses. A general review. Diagn. Microbiol. Infect. Dis..

[44] Lomate G.B., Dandi B., Mishra S. (2018). Development of antimicrobial LDPE/Cu nanocomposite food packaging film for extended shelf life of peda. Food Pack. Shelf Life.

[45] Li Z., Sun J., Lan J., Qi Q. (2016). Effect of a denture base acrylic resin containing silver nanoparticles on Candida albicans adhesion and biofilm formation. Gerodontology.

[46] Nanda A., Saravanan M. (2009). Biosynthesis of silver nanoparticles from Staphylococcus aureus and its antimicrobial activity against MRSA and MRSE. Nanomed. Nanotechnol. Biol. Med..

[47] Ziąbka M., Menaszek E., Tarasiuk J., Wroński S. (2018). Biocompatible nanocomposite implant with silver nanoparticles for otology—In vivo evaluation. Nanomaterials.

[48] Chen X., Lei H., Xu T., Zhang J., Qiu Y., Tan L. (2017). Preparation of PMMA/Nano-Cu antibacterial plastic by in situ polymerization. Key Eng. Mater..

[49] Cao J., Zhang W.X. (2006). Stabilization of chromium ore processing residue (COPR) with nanoscale iron particles. J. Hazard. Mater..

[50] Wang D., Lin Z., Wang T., Yao Z., Qin M., Zheng S., Lu W. (2016). Where does the toxicity of metal oxide nanoparticles come from: The nanoparticles, the ions, or a combination of both?. J. Hazard. Mater..

[51] EU Commission Regulation No. 10/2011 of 14 January 2011. https://eur-lex.europa.eu/legal-content/EN/TXT/PDF/?uri=CELEX:32011R0010&from=EN.

[52] Cushen M., Kerry J., Morris M., Cruz-Romero M., Cummins E. (2014). Silver migration from nanosilver and a commercially available zeolite filler polyethylene composites to food simulants. Food Addit. Contam. Part A.

[53] Bott J., Störmer A., Franz R. (2014). Migration of nanoparticles from plastic packaging materials containing carbon black into foodstuffs. Food Addit. Contam. Part A.

[54] Ozaki A., Kishi E., Ooshima T., Hase A., Kawamura Y. (2016). Contents of Ag and other metals in food-contact plastics with nanosilver or Ag ion and their migration into food simulants. Food Addit. Contam. Part A.

[55] Hauri J.F., Niece B.K. (2011). Leaching of silver from silver-impregnated food storage containers. J. Chem. Educ..

[56] Khalid M., Hassani S., Abdollahi M. (2020). Metals-induced oxidative stress: An evidence-based update of advantages and disadvantages. Curr. Opin. Toxicol..

[57] Sharifan H., Moore J., Ma X. (2020). Zinc oxide (ZnO) nanoparticles elevated iron and copper contents and mitigated the bioavailability of lead and cadmium in different leafy greens. Ecotoxicol. Environ. Saf..

[58] Loyo-Rosales J.E., Rosales-Rivera G.C., Lynch A.M., Rice C.P., Torrents A. (2004). Migration of nonylphenol from plastic containers to water and a milk surrogate. J. Agric. Food Chem..

